# Next-Generation Wound Healing Materials: Role of Biopolymers and Their Composites

**DOI:** 10.3390/polym17162244

**Published:** 2025-08-19

**Authors:** Jonghyuk Park, Ranjit De

**Affiliations:** School of Life Science, Handong Global University, Pohang 37554, Republic of Korea

**Keywords:** polymers, composites, skin regeneration, wound healing, biopolymers

## Abstract

The progress in biopolymers and their composites as advanced materials for wound healing has revolutionized therapeutic approaches for skin regeneration. These materials can effectively integrate their inherent biocompatibility and biodegradability with the enhanced mechanical strength and customizable properties of polymers and functional additives. This review presents a detailed investigation of the design principles, classifications, and biomedical applications of biopolymeric composites, focusing on their capabilities to promote angiogenesis, exhibit antimicrobial activities, and facilitate controlled drug delivery. By overcoming the challenges of conventional wound dressings, such as inadequate exudate management, mechanical fragility, and cytotoxicity, these composites provide dynamic, stimuli-responsive platforms that can adapt to the wound microenvironment. This study further highlights innovative advances in nanoparticle-assisted reinforcement, fiber-based scaffolds, and multi-stimuli responsive smart delivery systems. Finally, the future perspective illustrates how the challenges related to long-term physiological stability, scalable manufacturing, and clinical implementation can be addressed. Overall, this article delivers a comprehensive framework for understanding the transformative impact of biopolymeric composites in next-generation wound care.

## 1. Introduction

Skin is the largest organ in our body and functions as a vital protective barrier. It sustains metabolism, thermoregulation, and immune response. Therefore, maintaining skin health is fundamental to physiological function and resilience. Any wound in the skin that is fundamentally a disruption of tissue continuity caused by physical, chemical, thermal, immunological, or microbial attacks can impair structural integrity and function. If unaddressed, such injuries can lead to persistent pain, inflammation, infection, and potentially organ failure. Such wounds are broadly classified as acute and chronic. Acute wounds typically progress orderly through healing phases and are resolved within weeks without significant scarring, while chronic wounds, like diabetic foot ulcers, do not follow that same path and are often prolonged in an inflammatory state. This disruption in sequential healing, such as hemostasis, inflammation, proliferation, and remodeling, creates a major medical burden, causing delayed recovery, increased infection risk, and high healthcare costs. The complex pathophysiology of chronic wounds, marked by persistent inflammation, elevated proteinases, hypoxia, and bacterial accumulation, highlights an urgent need for better therapeutic strategies and advanced wound management materials [1,2,3].

**Figure 1:**
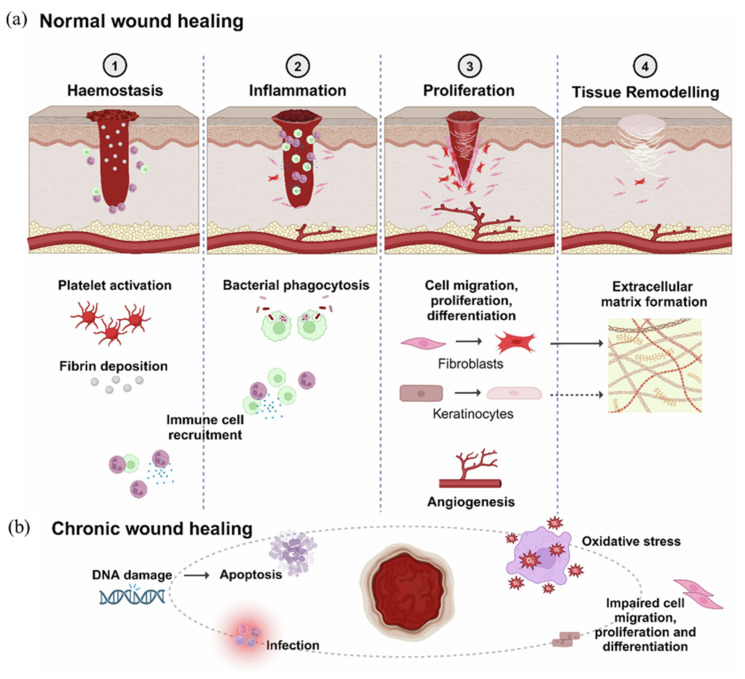
(**a**) Normal wound healing proceeds through four distinct phases: (1) hemostasis, where platelet activation and fibrin deposition prevent bleeding and initiate immune recruitment; (2) inflammation, where neutrophils and macrophages infiltrate the wound site, removing debris and secreting cytokines and growth factors; (3) proliferation, where fibroblasts, keratinocytes, and endothelial cells promote ECM formation, re-epithelialization, and angiogenesis; and (4) remodeling, where type III collagen is replaced with type I collagen, increasing tensile strength and completing scar formation. (**b**) Chronic wound healing is characterized by a prolonged inflammatory phase due to cytokine imbalance, microbial infection, excessive ROS production, and impaired angiogenesis. This results in delayed tissue regeneration, apoptosis, and dysfunction in cell proliferation, migration, and differentiation. Reprinted from [4] under Creative Commons License CC-BY 4.0.

Wound healing is the process of restoring the structure and function of damaged tissue. General wound healing involves four phases, hemostasis, inflammation, proliferation, and remodeling, and it is a highly dynamic and complex biological process [4] (Figure 1a). Each phase involves the interaction of various molecules, including different cells, signaling molecules, and the extracellular matrix (ECM) [5,6,7]. Understanding these wound healing processes is critical to designing and applying biopolymeric composites that can directly participate in or facilitate these biological processes. (1) Hemostasis phase. When a wound forms and bleeds, the body initiates a hemostatic response to stop the bleeding. Platelets gather at the wound site, release clotting factors, and activate the coagulation chain reaction to form a clot composed of fibrin. This clot stops bleeding and supports immune and tissue cell migration [8]. (2) Inflammatory phase. The inflammatory phase usually lasts for several days. During this phase, immune cells, such as neutrophils and macrophages, infiltrate the wound site. Neutrophils remove pathogens and cellular debris through phagocytosis. At the same time, macrophages secrete inflammatory cytokines, such as IL-1β and TNF-α, and growth factors, such as VEGF and TGF-β, to prepare tissue for regeneration. This is a critical time to control infection and start regeneration [9]. (3) Proliferation phase. This phase is characterized by the formation of new tissue. Fibroblasts migrate to the wound site and produce collagen and extracellular matrix (ECM) proteins to form the structural basis of new tissue. At the same time, endothelial cells restore oxygen and nutrient supply through angiogenesis. Keratinocytes begin the process of re-epithelialization, where they proliferate and migrate to re-cover the wound site [10]. (4) Remodeling or maturation phase. This final stage can last from a few weeks to a few months, depending on the type of wound. During this phase, the type III collagen formed during the proliferation phase is replaced by stronger type I collagen, increasing the tissue’s tensile strength. Myofibroblasts induce wound contraction, and matrix metalloproteinases (MMPs) break down excess ECM. The result is scar formation. Abnormal scarring can occur if these steps are not properly regulated. In conclusion, for successful healing to occur, each step must occur at the proper time and sequence [11]. However, this process is interrupted or delayed in chronic wounds, mainly during the inflammatory phase, due to an imbalance in the cytokine environment. The cytokine milieu is characterized by persistent microbial infection, persistent infiltration of neutrophils and macrophages, and increased pro-inflammatory cytokines (e.g., IL-1β, TNF-α). This dysregulated immune response leads to prolonged inflammation, excessive production of reactive oxygen species (ROS), overexpression of proteolytic enzymes, such as matrix metalloproteinases (MMPs), and impaired angiogenesis, ultimately impeding progression to the proliferative and remodeling phases of healing [3,4,12,13] (Figure 1b). To deal with delayed wound healing, biopolymeric composites can be designed to have properties such as antimicrobial functionality, controlled drug release, oxygen permeability, and mechanical support, which can contribute to supporting or facilitating each stage of healing [14,15].

**Figure 2:**
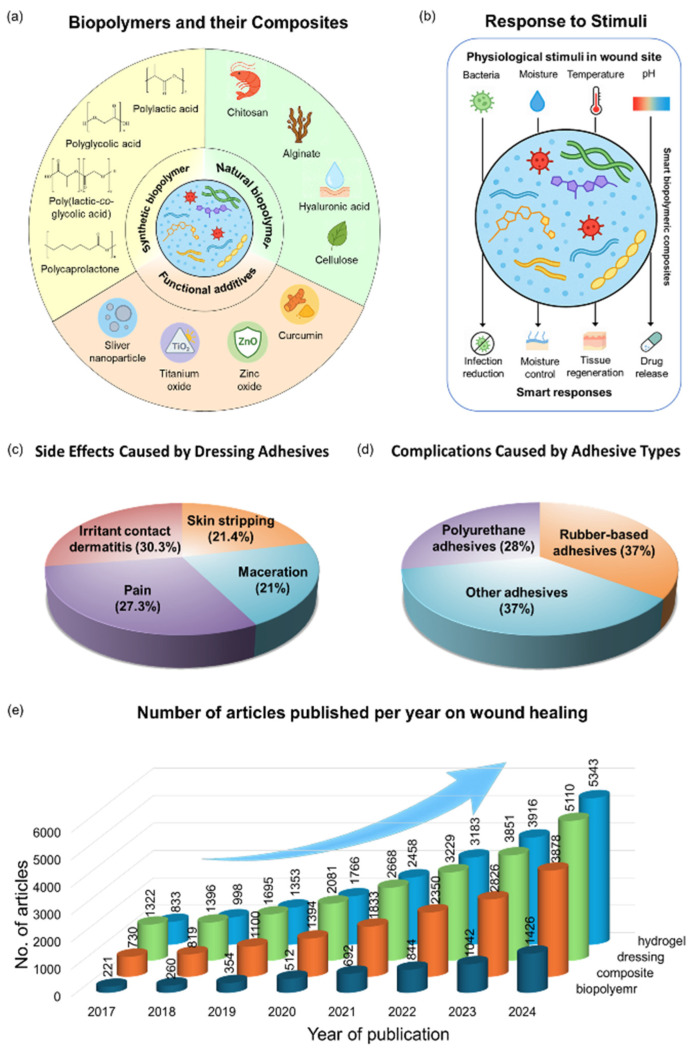
(**a**) Smart biopolymeric composites are composed of three main components: natural biopolymers (e.g., chitosan, alginate, hyaluronic acid, cellulose), synthetic biopolymers (e.g., PLA, PGA, PLGA, PCL), and functional additives (e.g., silver nanoparticles, titanium dioxide, zinc oxide, curcumin). Each component contributes to the overall functionality of the composite, allowing structural support, bioactivity, and enhanced therapeutic potential. (**b**) Smart biopolymeric composites respond to specific physiological stimuli, such as pH, temperature, moisture, and bacterial presence, by triggering tailored therapeutic responses, including controlled drug release, antimicrobial activity, tissue regeneration, and moisture regulation. This adaptive behavior enables effective and context-sensitive wound healing. (**c**) Distribution of the most frequently reported side effects associated with dressing adhesives, including pain (30%), irritant contact dermatitis (27%), maceration (21%), and skin stripping (21%). (**d**) Proportion of complication rates associated with different adhesive types, with rubber-based adhesives (35%) and polyurethane adhesives (28%) showing the highest incidence. Data obtained from [29]. (**e**) Number of articles published per year on wound healing (database: Scopus). Keywords used for the searches included “wound healing” and then each of the following: “hydrogel”; “dressing”; and “composite”.

Modern advances in caring for wounds, including chronic ones, have attracted substantial attention toward the smart biopolymeric composites as next-generation materials for promoting effective and expedited healing [16]. These multifunctional composite materials are engineered by combining the natural advantages of biopolymers, such as chitosan, alginate, collagen, hyaluronic acid, etc., with the structural and functional advantages of synthetic polymers and their nanoformulations [17,18,19]. As a result, such materials demonstrate not only biocompatibility and biodegradability but also respond intelligently to numerous physiological stimuli, including variations in pH, temperatures, magnetism, moisture levels, or the presence of enzymes and bacteria. Such sensitivity, along with selectivity, has expanded the prospect of developing smart systems appropriate for real-time adaptation to the wound milieu, allowing controlled drug delivery, reduced infection risk, and optimized moisture balance (Figure 2a,b) [20,21]. In contrast to conventional wound dressings, which often provide passive defense, smart biopolymeric composites offer dynamic and targeted healing support, promoting tissue regeneration, angiogenesis, and inflammatory regulation. Their tunable properties also make them ideal platforms for integrating antimicrobial agents, growth factor proteins, and stem cells [22,23,24,25]. Conventionally used synthetic materials, such as polyurethane, polyethylene glycol, silicone, etc., have been widely employed in wound healing applications. While these dressings assist by maintaining a moist environment favorable for healing, they often fail to effectively absorb the wound exudate. Moreover, their use can be linked to several harmful effects, which include increased risk of infection, excessive fluid retention, and potential tissue necrosis [26,27,28]. In a recent study, approximately 27% of surgical patients were reported to experience complications associated with dressing adhesives. The most frequently observed side effects included pain (27.3%), irritant contact dermatitis (30.3%), maceration (21%), and skin stripping (21.4%). Among various adhesive types, rubber-based adhesives (37%) and polyurethane adhesives (28%) were associated with the highest incidence of complications (Figure 2c,d) [29]. Biopolymers, on the other hand, are gaining increasing attention due to their superior exudate management, moisture regulation, biocompatibility, and biodegradability. These are naturally occurring or synthetically produced polymers composed of repeating monomeric units derived from living organisms or biological sources. They are categorized based on their source of origin, chemical composition, and biosynthetic pathway, and they can broadly be classified into natural, synthetic, and semi-synthetic biopolymers [30,31]. Being sourced from living organisms, including plants, animals, microorganisms, etc., natural biopolymers are typically both biocompatible and biodegradable, with widely investigated examples including chitosan, alginate, cellulose, and collagen [16,32,33]. Synthetic biopolymers are obtained through chemical polymerization of biological monomers or through biotechnological methods, such as microbial fermentation. They offer tunable physical and chemical properties. Examples of such biopolymers are polylactic acid (PLA), polyglycolic acid (PGA), poly(lactic-co-glycolic acid) (PLGA), and polycaprolactone (PCL) [34,35]. A biopolymeric composite material is engineered by combining two or more different constituents, typically consisting of a matrix composed of polymer, metal, or ceramic with reinforcing elements like fibers, particles, or functional additives [36]. Since these biopolymeric materials are highly valued in biomedical fields for their superior biocompatibility and biodegradability, they are often formulated as combinations of different biopolymers and various materials that possess distinct chemical or physical properties [37,38]. Occasionally, biopolymers used in isolation undergo degradation or nonfunctional modifications that impair their intended biological or mechanical performance. Environmental factors such as heat, pH, light, etc., can negatively affect their stability and function, both internally and externally [39,40]. To mitigate these issues, strategies involving material modification or blending are often employed to enhance their functional stability. Thereby, biopolymeric composites bear the potential to become promising candidates suitable for overcoming the functional limitations of conventional biopolymers. By combining biopolymers with synthetic or natural reinforcements, their physical properties are enhanced, resulting in controlled degradation rates and improved efficacy for a wide range of biomedical applications [41,42,43]. Such modified biopolymeric composites can improve not only physical properties but also enable the integration of physiologically active agents that promote therapeutic functions. Thus, the overall properties of the biopolymeric composites get improved and subsequently can be used in various biomedical applications. Specifically, by binding with multifunctional additives, biopolymeric composites can simultaneously offer both biodegradability and antibacterial properties, which is highly beneficial for addressing key challenges in wound care [44,45,46]. Based on therapeutic approaches, wound treatment is broadly categorized into “traditional” and “regenerative” approaches. Traditional treatment consists of infection control and autologous skin grafts, but it has disadvantages such as multiple surgeries, limited donor sites, and scarring. Regenerative treatments, on the other hand, aim to achieve scar-free healing and restore skin function through smart dressings, biomaterials, stem cell and gene therapy, and bionic skin grafts [47,48,49]. For example, in treating the skin of a burn patient, traditional methods require harvesting skin from healthy areas for grafting, which causes additional scarring and limits the availability of healthy tissue, whereas regenerative treatment utilizes stem cell-based artificial skin to overcome these limitations [50]. Some biomaterials can also exhibit synergistic effects, which not only support tissue regeneration but also simultaneously reduce inflammatory responses and the risk of infections, which is favorable for achieving both the “traditional” and “regenerative” therapeutic effects. As a result, there is a growing demand for sustainable and effective biopolymeric composites for wound healing applications [51,52]. In 2023, the global biomaterial wound dressings market was valued at USD 6.23 billion. It is expected to grow at a compound annual growth rate (CAGR) of 7.46% between 2024 and 2030, reaching USD 10.31 billion [53]. Correspondingly, the increase in investigations about biopolymeric composites for wound healing is noticeable through the growing number of studies, as presented in Figure 2e.

Given the rapid advancements in biomaterials science, a thorough understanding of biopolymeric composites is essential for the development of next-generation smart healthcare materials. This review focuses on the emerging role of biopolymeric composites in wound healing and their broader applications in the biomedical field. These materials combine the advantages of natural biopolymers, such as biocompatibility and biodegradability, with enhanced functional properties offered through composite design. This study explores current strategies used for designing these composites, emphasizing recent innovations aimed at improving therapeutic outcomes in wound care. It also addresses the limitations of conventional biopolymers and how composite approaches can overcome these challenges. By highlighting key developments and emerging trends, this overview examines the ongoing research efforts to engineer materials that promote tissue regeneration, reduce healing time, and prevent infection. Furthermore, it offers a practical guide for selecting suitable biopolymers for wound healing applications. Finally, the review presents the future potential of these advanced materials and the challenges in translating them into clinical practice.

## 2. Biopolymers for Wound Healing

### 2.1. Natural Biopolymers and Their Significance in Wound Healing

Biopolymers are high molecular weight compounds synthesized by living organisms, including polysaccharides, proteins, nucleic acids, and lipids, each contributing critically to fundamental biological processes. They are characterized by unique molecular architectures and functions. Examples of some common biopolymers include cellulose, starch, chitin, collagen, and silk [54,55]. Along with biodegradability and biocompatibility, these biopolymers are also notable due to some of their inherent properties, such as non-toxicity, renewable origin, and cost-effectiveness, which make them promising candidates for sustainable material design. Their biodegradability ensures environmental benevolence by natural degradation processes, while biocompatibility supports safe use in numerous biological and medical applications [32,56,57,58]. In addition to their biological significance, natural biopolymers exhibit diverse physicochemical properties, including the ability to form film, demonstrate mechanical strength, and allow easy chemical modification, which contribute to their ever-expanding use across a wide range of industries [59,60]. They are increasingly employed in food packaging as eco-friendly replacements to synthetic plastics, in medicine for drug delivery, wound healing, and tissue engineering scaffolds. They have also gained significant interest in agriculture for the controlled use of fertilizers and soil conditioners. Furthermore, recent technological advancements have attracted significant attention to designing biopolymer composites, derivatives, and nanostructures to enhance their performance and application scope, reflecting their growing importance as sustainable next-generation healthcare materials [61,62,63,64]. Therefore, understanding the fundamental characteristics and diverse applications of natural biopolymers is essential for advancing their development and addressing global environmental issues. The significance of certain naturally occurring biopolymers is discussed below.

#### 2.1.1. Alginate for Multifunctional and Biocompatible Wound Healing Material

**Figure 3:**
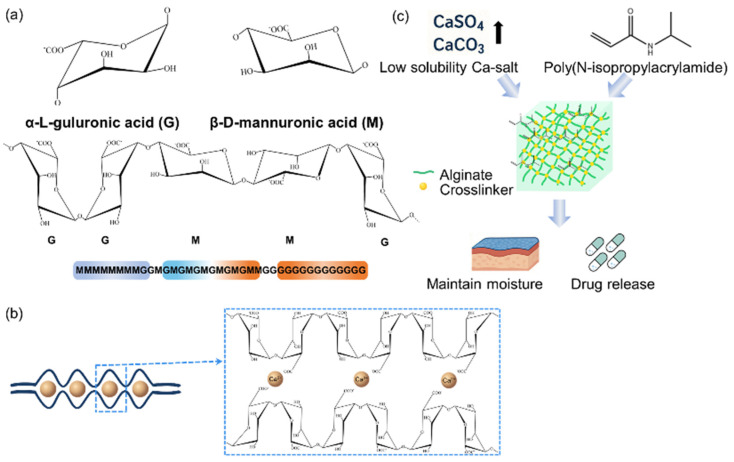
(**a**) Alginate is composed of β-D-mannuronic acid (M) and α-L-guluronic acid (G), forming homopolymeric (MM, GG) and heteropolymeric (MG, GM) blocks. The M/G ratio and sequence distribution critically influence gelation behavior and mechanical properties. (**b**) Divalent calcium ions (Ca^2+^) interact with carboxyl groups of G-blocks to form ionic bridges, resulting in a stable hydrogel. (**c**) Calcium salts with low solubility (CaSO_4_, CaCO_3_) slow the gelation rate; incorporation of PNIPAAm imparts thermo-responsive behavior. The resulting hydrogel maintains a moist environment and allows controlled drug release.

Alginate, which is a linear, unbranched polysaccharide, is predominantly extracted from brown seaweed. Notably, alginate is an anionic polysaccharide composed of two types of monomers: α-(1→4)-linked L-guluronic acid (G) and β-(1→4)-linked D-mannuronic acid (M). These monomers are arranged in either homo blocks (MM or GG) or hetero blocks (MG or GM) (Figure 3a). The M/G ratio, block length, and molecular weight are key factors that determine the physical properties of alginate [65,66,67,68]. These polymers are known for their excellent biocompatibility. Studies have shown that high-purity alginates trigger lower immune responses, making them safer for in vivo applications. Torres et al. reported that unpurified alginate contains impurities, such as polyphenols and proteins, which can trigger an immune response, leading to cytotoxicity and inflammation. However, a simple purification process can reduce the immune response and thereby improve biocompatibility and osteoinductivity. For example, Torres et al. have reported that 1% alginate solution can be treated with a chloroform/butanol mixture (4:1 by volume) to remove protein impurities, followed by ethanol precipitation, freeze-drying, and a 7-day dialysis process to remove salts and residual contaminants. This process effectively removes immunogenic components, such as polyphenols, proteins, and endotoxins, thereby preventing these components from activating macrophages, stimulating nitric oxide release, and inducing inflammatory cytokine production, thus minimizing inflammatory activation [69]. Alginate also has gelation ability. Physical and chemical properties depend on the gelation method: ionic crosslinking, covalent bonding, and thermal gelation. Especially, only G-blocks participate in crosslinking with divalent cations (Ca^2+^, etc.) to form hydrogels, creating a three-dimensional structure that retains water (Figure 3b) [70,71]. Mechanical properties of these gels can be strengthened by slowing down the gelation rate. Due to relatively low solubility, calcium sulfate (CaSO_4_) and calcium carbonate (CaCO_3_) can also contribute to slowing down the gelation rate [72]. It has been observed that crosslinking using CaSO_4_ significantly improved the mechanical properties of the polymer gel by increasing the viscosity by 100-fold from about 0.4 Pa s to 40.4 Pa s and enhancing the storage modulus G′ to 219 Pa. Furthermore, the gelation rate was slowed, leading to increased structural uniformity, while high cell viability was maintained as observed by Kostenko et al. [73]. However, ionically crosslinked alginate gels are unlikely to have long-term stability under physiological conditions, as divalent cations can be exchanged with monovalent cations, causing the gel to degrade. At this point, covalent crosslinking can improve the mechanical strength and stability of alginate gels. Furthermore, various crosslinking agents and crosslinking methods, including photo-crosslinking, can show precise control properties and degradation rates [74,75]. Araiza-Verduzco et al. reported that the compressive strength of photopolymerized alginate–methacrylate hydrogels (H-ALGMx) depended on the methacrylate introducing group, with ALGM3-derived gels exhibiting the highest compressive strength (maximum σ_x_ ≈ 38.5 kPa) and toughness (UT ≈ 5.9 kJ m^−3^), indicating the best mechanical performance. This was attributed to the molecular structure, where the C=C bonds are arranged outwardly with respect to the polymer chain and are free to rotate, allowing photopolymerization crosslinking to occur more efficiently [76]. Nevertheless, the toxicity and removal of the residual crosslinking agents have often been a major challenge. Heat-sensitive hydrogels, such as poly(n-isopropylacrylamide) (PNIPAAm)-based hydrogels, are useful for drug delivery because they can swell with temperature changes and thereby control drug release [77]. Alginates are inherently insensitive to heat. However, thermo-sensitive hydrogels with improved mechanical strength and drug release can be developed by reacting PNIPAAm with alginates or forming a semi-permeable structure (semi-interpenetrating polymer network, semi-IPN) [78,79]. Alginate has a high-water absorption capacity and can maintain a moist wound environment while managing excess exudate, which can be exploited to optimize conditions for wound healing [80]. Savić et al. have shown that the optimized alginate hydrogel (2.7% alginate, 0.9% CaCl_2_) showed a 76.0% water retaining capacity, 94.3% moisture content, and a swelling ratio of 9.4 g/g, supporting its suitability for moist wound environments [81]. These properties make alginate suitable for designing wound healing hydrogels (Figure 3c).

#### 2.1.2. Chitosan for Antibacterial Protection and Bio-Responsive Healing

**Figure 4:**
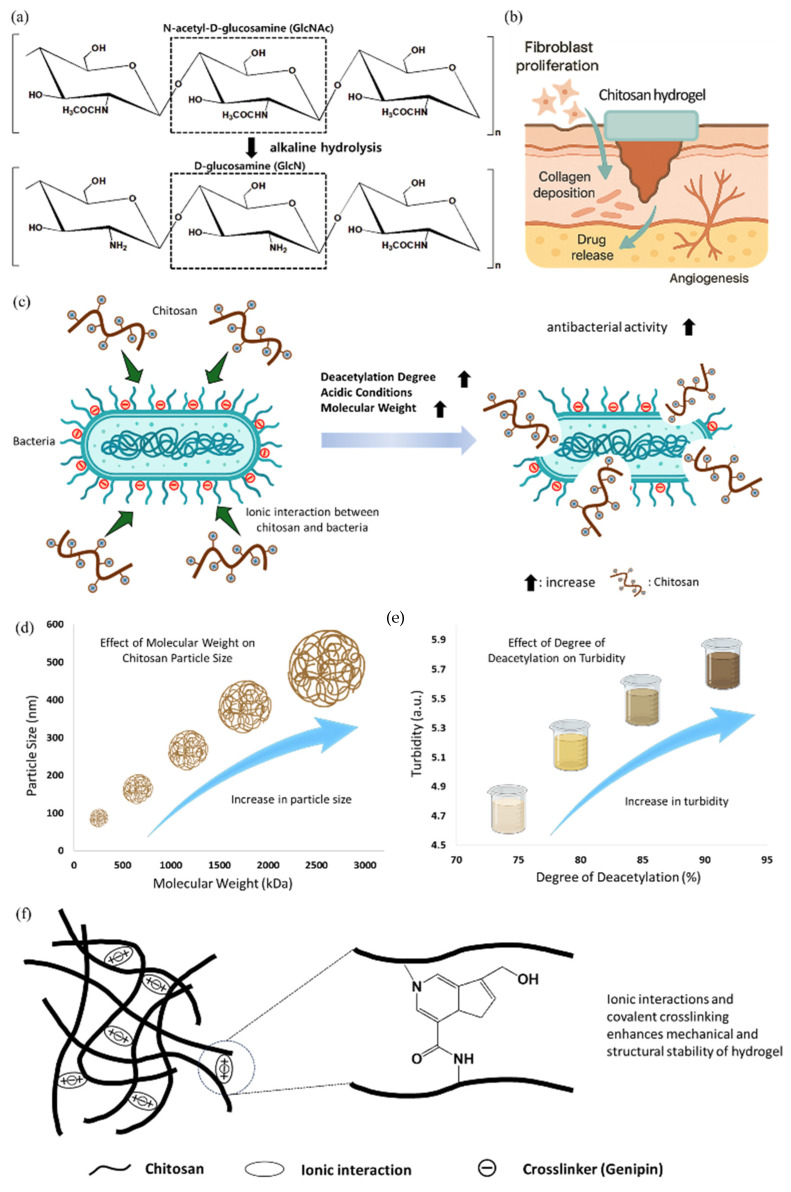
(**a**) Alkaline hydrolysis removes acetyl groups from N-acetyl-D-glucosamine (GlcNAc) units, producing a copolymer of D-glucosamine (GlcN) and N-acetyl-D-glucosamine (GlcNAc). (**b**) Schematic illustration of the enhanced antibacterial activity of chitosan. Under acidic conditions, chitosan becomes positively charged and interacts with negatively charged bacterial surfaces. Increasing the degree of deacetylation and molecular weight further enhances these electrostatic interactions, leading to improved antibacterial effects. (**c**) Chitosan promotes fibroblast proliferation, collagen deposition, drug release, and angiogenesis, facilitating accelerated wound healing. (**d**) Effect of molecular characteristics on chitosan properties. Increasing the molecular weight of chitosan from 81 kDa to 3000 kDa leads to a significant increase in particle size. (**e**) Raising the degree of deacetylation from 77% to 89% increases turbidity, indicating enhanced charge density and intermolecular interactions. (**f**) Chitosan chains form networks via ionic interactions and covalent crosslinking with crosslinker (genipin), enhancing the mechanical and structural stability of the hydrogel.

Chitosan is a naturally occurring polysaccharide obtained by the partial deacetylation of chitin, which is the most abundant biopolymer on earth after cellulose. Chitin is found primarily in the exoskeletons of crustaceans, such as shrimps and crabs, fungi cell walls, and insect cuticles [82,83]. Through alkaline hydrolysis, chitin is converted to chitosan by removing the acetyl group from the N-acetyl-D-glucosamine (GlcNAc) unit, resulting in the formation of a copolymer composed of D-glucosamine (GlcN) and N-acetyl-D-glucosamine (Figure 4a) [84]. Chitosan exhibits several desirable biological properties, including biocompatibility, biodegradability, and non-toxicity. These properties make chitosan suitable for biomedical applications, especially in wound healing. Chitosan facilitates wound healing by promoting fibroblast proliferation, supporting collagen deposition, and enhancing angiogenesis (Figure 4b). The degree of deacetylation (DD) is an important factor affecting solubility, charge density, and biological activity [85]. Amor et al. showed that chitosan with 88.2% DD had superior antibacterial activity and dye adsorption (1.7 mg g^−1^) under neutral pH [86]. The observed increase in antibacterial activity and surface functionality at higher DD suggests indirect relevance to infection control in wound dressings. Chitosan is positively charged under acidic conditions due to protonation of the amine groups, which enables electrostatic interactions with negatively charged surfaces, including bacterial membranes and extracellular matrix components (Figure 4c) [87,88]. Chitosan can also form films and hydrogels, acting as drug carriers to maintain a moist wound environment and release topical therapeutics [89,90]. The physicochemical properties of chitosan, including viscosity, mechanical strength, porosity, and degradation rate, can be controlled by adjusting the molecular weight, degree of deacetylation, and crosslinking strategy [91]. By increasing chitosan’s molecular weight from 81 kDa to 3000 kDa, the particle size was found to increase from 363 ± 37 nm to 539 ± 32 nm (Figure 4d). Similarly, raising the degree of deacetylation from 77% to 89% elevated turbidity from 4.87 to 5.98, indicating higher charge density and stronger interactions, as reported by Wang et al. (Figure 4e) [92]. Furthermore, in another study by Dutta et al., it has been understood that at 1% concentration, chitosan with ~80% DD showed the highest Water Vapor Transmission Rate (WVTR) (~2300 gm^−2^ per day) and water absorption (~197%), with a contact angle of 76.76°, indicating superior hydrophilicity [93]. Crosslinking agents such as genipin, glutaraldehyde, and ionic compounds are commonly used to improve the mechanical stability of chitosan-based hydrogels or scaffolds (Figure 4f) [94]. Chitosan can also be chemically modified through grafting or functionalization to introduce stimulus-responsive behaviors, including pH- or temperature-sensitive drug release [89]. Despite its many advantages, chitosan has limitations, such as low solubility at physiological pH and relatively weak mechanical strength when used alone. Therefore, chitosan is often blended with other natural or synthetic polymers such as gelatin, alginate, or polyvinyl alcohol to improve its structural and functional performance in biomedical applications [95,96]. Thus, due to its broad functionality and ease of modification, chitosan is one of the most extensively studied natural polymers in wound dressings and controlled drug delivery.

#### 2.1.3. Hyaluronic Acid for Gel Design with Excellent Water Retention Capacity

**Figure 5:**
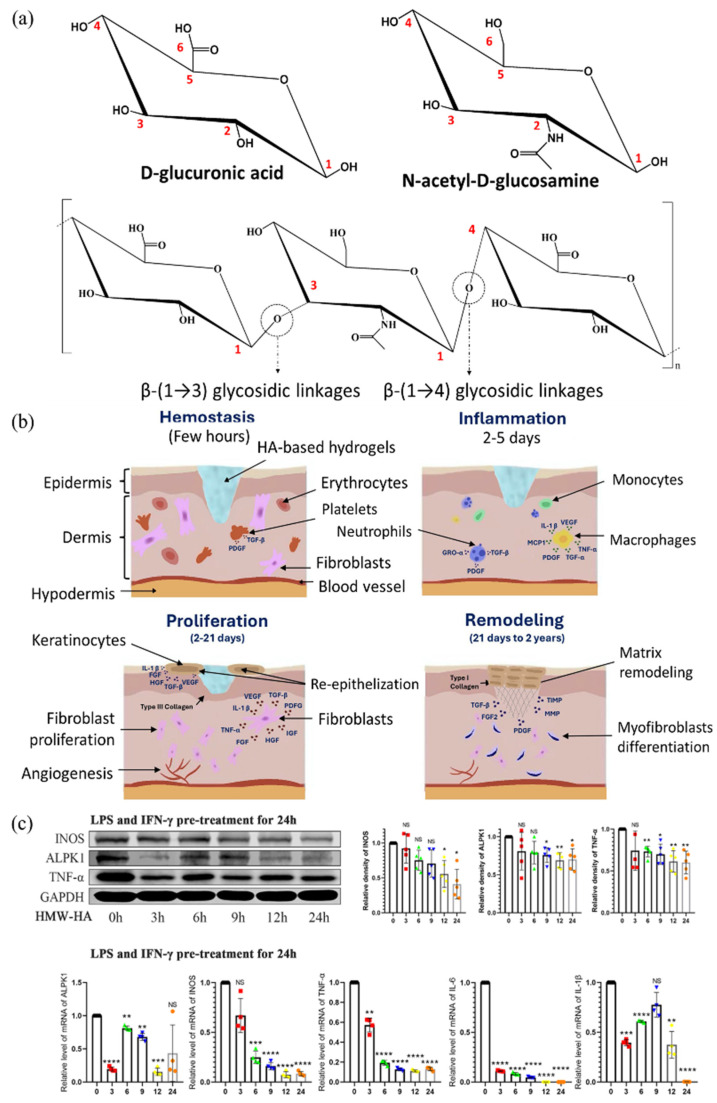
(**a**) Structures of D-glucuronic acid and N-acetyl-D-glucosamine and the repeating unit of hyaluronic acid with β-(1→3) and β-(1→4) glycosidic linkages. (**b**) HA contributes to wound healing by supporting hemostasis, regulating inflammatory responses, promoting fibroblast proliferation and angiogenesis, and facilitating extracellular matrix remodeling, depending on its molecular weight and structural properties. Reprinted from [101] under Creative Commons License CC-BY 4.0. (**c**) Following LPS and IFN-γ stimulation, treatment with high molecular weight hyaluronic acid (1500–2500 kDa) reduced both protein and mRNA levels of ALPK1 (inflammatory sensor), INOS (nitric oxide synthase), TNF-α, IL-6, and IL-1β (pro-inflammatory cytokines) over time, suggesting inhibition of M1 macrophage polarization. Reprinted from [112] under Creative Commons License CC-BY 4.0.

Hyaluronic acid (HA) is a naturally occurring polysaccharide widely found in the human body, especially in the skin, synovial fluid, the eye’s vitreous humor, cartilage, and connective tissue. Alternatively, HA can be produced through microbial fermentation using bacteria, such as *Streptococcus* species [97]. HA is composed of repeating disaccharide units of D-glucuronic acid and N-acetyl-D-glucosamine linked by alternating β-(1→4) and β-(1→3) glycosidic linkages (Figure 5a) [98]. In the physiological environment, HA carries a net negative charge. In the body, it is found at various molecular weights, such as 770–1700 kDa in the vitreous humor, 6000–7000 kDa in normal synovial fluid, and 3000–5000 kDa in rheumatoid synovial fluid, depending on the tissue type. HA influences biological functions throughout the different stages of wound healing. It is highly variable in size and significantly impacts wound healing [99,100,101]. Polymeric HA primarily contributes to structural integrity by filling the extracellular space and retaining tissue hydration. At the same time, low molecular weight HA fragments play an important role in wound healing by transmitting cell signaling, triggering inflammatory responses, and promoting cell migration and proliferation (Figure 5b) [101,102]. HA has excellent viscoelasticity, which provides high moisturizing efficacy and biocompatibility. Despite low concentrations, HA exhibits high viscosity due to its remarkable viscoelastic properties. It can act as a lubricant, shock absorber, joint stabilizer, water balance, and flow resistance regulator [103]. Rananathan et al. reported that high-MW HA gels at 0.7% (*w*/*v*) exhibited high viscosities ranging from 145 to 300 mPa, supporting their inherent viscoelasticity and suitability for biomedical applications [104]. On average, there are about 15 g of HA in the body of a 70 kg adult. Located in joints, skin, eyes, connective tissue, epithelium, and nervous tissue, approximately 5 g of HA is replaced per day [105,106]. HA is synthesized by hyaluronan synthase (HAS1, HAS2, HAS3), which produces HA of different sizes. It is lengthened by repeated addition of glucuronic acid and N-acetylglucosamine. Degradation has occurred due to hyaluronidase, and a decrease in HA molecular weight is observed in advanced cases [107,108]. HA has physiological functions to maintain viscoelasticity and aid in tissue lubrication and regeneration, as well as pharmacological functions of antioxidant and anti-inflammatory effects, immunomodulation, joint protection, and analgesia. These beneficial effects are dependent on its molecular weight [109,110]. In osteoarthritic synoviocytes, high molecular weight HA (1000–2000 kDa) can significantly reduce IL-6 and PGE2 levels and promote M2 macrophage polarization via downregulation of GRP78 and NF-κB signaling [111]. Zhao et al. reported that low molecular weight HA (10–100 kDa) significantly increased the expression of ALPK1, TNF-α, IL-6, IL-1β, and INOS, while high molecular weight HA (1500–2500 kDa) suppressed their expression and inhibited nuclear translocation of PKM2, thereby modulating macrophage polarization in mouse tumor (RAW264.7) cells (Figure 5c) [112]. These findings highlight the molecular weight-dependent immunomodulatory roles of HA. Thus, HA promotes wound healing by providing structural support and stimulating cellular activity essential for tissue regeneration.

#### 2.1.4. Cellulose for Hydrogel with Improved Mechanical Stability

**Figure 6:**
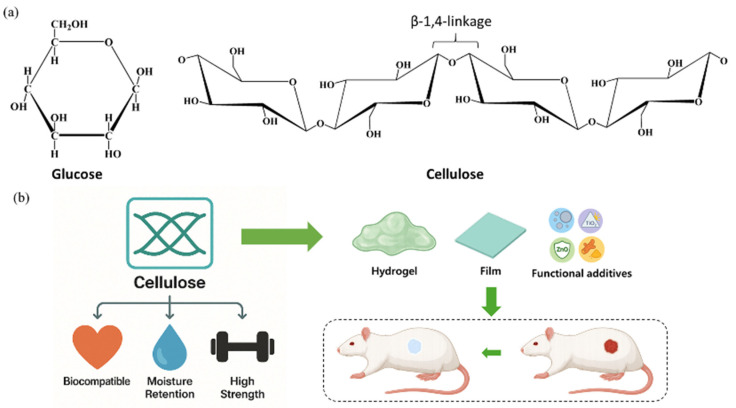
(**a**) Structure of glucose monomer and cellulose polymer linked via β-1,4-glycosidic bonds. (**b**) Cellulose exhibits biocompatibility, moisture retention, and mechanical strength, enabling its transformation into hydrogels and films with functional additives.

Cellulose is a fundamental structural biopolymer predominantly found in plant cell walls, and it is synthesized in vast quantities globally, with production estimates reaching billions of tons each year [113,114]. Beyond its biological abundance, cellulose contributes significantly to the global carbon cycle by participating in atmospheric carbon dioxide sequestration. While it is mainly associated with plants, cellulose is also produced by a broad range of organisms such as bacteria, protozoa, algae, and some invertebrate animals [115,116]. Notably, cyanobacteria, which are believed to have appeared over 3.5 billion years ago, are among the earliest known cellulose producers. Across all producing species, cellulose is secreted into the extracellular matrix, where it performs structural roles [117]. On a molecular level, cellulose is classified as a β-1,4-linked glucan composed of repeating glucose units (Figure 6a) [118]. These glucose monomers are connected via acetal bonds between the C1 and C4 carbon atoms, forming a linear polysaccharide chain. The β-anomeric configuration induces an alternating orientation of adjacent glucose residues, resulting in a repeating cellobiose unit [119]. Extensive intra- and inter-molecular H-bonding occurs, particularly involving the hydroxyl groups at the C2, C3, and C6 positions. These H-bonds stabilize the planar alignment of the glucose rings, producing a ribbon-like conformation with significant mechanical rigidity. The glycosidic bonds in cellulose exhibit remarkable chemical stability, with some estimated to persist for millions of years in natural conditions [119,120,121]. Additionally, cellulose’s biodegradable nature allows it to decompose into simple byproducts, such as water, carbon dioxide, and biomass, through microbial enzymatic degradation. This ecological compatibility makes cellulose a highly attractive candidate for applications in sustainable packaging and implantable biomaterials [122,123,124]. From a physicochemical standpoint, cellulose is highly hydrophilic due to its abundant hydroxyl functional groups, which allow it to absorb and retain large quantities of water [125]. In a study, Grolman et al. reported that treating deep partial-thickness porcine burn wounds with an agarose hydrogel reduced the depth of tissue necrosis by approximately 40% (227 μm vs. 358 μm) when a moist environment was maintained for four days immediately after injury [126]. So, the water retention capacity plays a crucial role in wound management, as it helps sustain a moist healing environment that supports tissue regeneration and minimizes scar formation. In the case of cellulose, Perdoch et al. found that cellulose exhibited a moisture content of 280% and a fiber saturation point (FSP) of 40.2 ± 1.6%. This result indicates its high hydrophilicity due to abundant hydroxyl groups, which facilitate significant water absorption and retention [127]. The fibrous structure of cellulose, coupled with its high crystallinity and hydrogen bonding network, also confers exceptional tensile strength, frequently exceeding 120 MPa, which is advantageous in biomedical and structural applications. In addition to its mechanical durability, cellulose offers thermal resilience, maintaining structural integrity under elevated temperatures [128,129,130]. These properties collectively render cellulose vital in developing composites, bioplastics, and various medical devices, such as surgical sutures and scaffolds. Its biocompatibility, moisture-regulating capacity, and mechanical performance contribute to its growing use in wound healing applications (Figure 6b).

### 2.2. Synthetic Biopolymers: Tunable Platforms for Biomedical and Therapeutic Applications

Synthetic biopolymers, meticulously engineered to emulate the structural and functional attributes of their natural counterparts, represent a growing class of materials in biomedical engineering. These biopolymers are typically synthesized via controlled chemical or biochemical processes, employing a diverse array of monomers to achieve desired properties [30,131]. In contrast to naturally occurring biopolymers, synthetic biopolymers offer enhanced tunability, allowing for precise modulation of their physicochemical characteristics to meet the demands of specific applications. Examples of synthetic biopolymers include poly(lactic acid) (PLA), poly(glycolic acid) (PGA), poly(lactic-co-glycolic acid) (PLGA), polycaprolactone (PCL), etc. [132,133,134]. The versatility of synthetic biopolymers, however, is challenged by potential biocompatibility concerns, necessitating careful consideration of non-toxic design strategies. Despite the potential biocompatibility challenges, the tunable properties of synthetic biopolymers have favored their increasing adoption in biomedical applications, particularly in wound healing. The ease of modifiable properties of these polymers to meet various needs opened new directions in which they could be effectively employed [5,18,135]. Synthetic polymers have garnered increasing interest as therapeutic agents, owing to their enhanced pharmacokinetic profiles relative to small-molecule drugs [136]. They can also be combined with synthetic polymers as reinforcement materials. This has prompted innovation across diverse biomedical fields. The prospect of using synthetic polymers as biomaterials has attracted ever-increasing attention in recent years [30,137]. Some of the applications of these synthetic biopolymers are summarized in Table 1.

#### 2.2.1. Polylactic Acid: Tunable for Antibacterial and Immunomodulatory Healing

PLA is a linear aliphatic thermoplastic polyester belonging to the poly(α-hydroxy acid) family, synthesized from lactic acid, which exists as L- and D-isomers. The ratio and arrangement of these isomers determine the stereoisomeric form of PLA, which affects its crystallinity, degradation rate, and mechanical/thermal properties. A racemic mixture of L- and D-type units produces amorphous or semi-crystalline PLA with controllable properties [138,139,140,141]. PLA is primarily synthesized via condensation polymerization or ring-opening polymerization. Condensation is cost-effective but requires high temperatures (180–200 °C), long reaction times, and organic solvents, making it inefficient for producing high molecular weight PLA [142,143,144]. By controlling the reaction time and conditions, chain extenders can be used to increase molecular weight [145]. Liu et al. reported that using hexamethylene diisocyanate as a chain extender and reacting at 175 °C for 20 min, the weight-average molecular weight of PLA increased from 2.0 × 10^4^ g mol^−1^ to 20.3 × 10^4^ g mol^−1^, demonstrating that molecular weight can be effectively enhanced by controlling reaction time and conditions [146]. Ring-opening is a process that enables the synthesis of high molecular weight PLA at low temperatures and in a short time using purified lactide [139,147,148]. By adjusting D-/L-lactide ratios, properties such as crystallinity, strength, and thermal stability can be precisely tuned [149,150,151]. In tensile tests, Pölöskei et al. reported that PLA films with 1.4% D-lactide showed crystallinity increases from 6.6% to 47.6% at 75 °C, whereas 12% D-lactide films remained amorphous [152]. Poly-L-lactic acid (PLLA) is highly crystalline with superior mechanical properties, while poly-D-lactic acid is less crystalline and weaker [149,150]. Horváth et al. reported that PLA made by ring-opening polymerization exhibited a Tg of 34.6 °C, cold crystallization at 76.4 °C, and a melting point of 110.3 °C, with 27.6% crystallinity [147]. Such tunability is crucial for tailoring PLA’s degradation and structural support properties in wound healing applications. Owing to its biocompatibility, biodegradability, and mechanical flexibility, PLA has been extensively studied as a scaffold material in skin tissue engineering. For example, electrospun PLA nanofibrous mats effectively mimic the extracellular matrix (ECM), supporting fibroblast and keratinocyte attachment, proliferation, and migration while preserving wound moisture and enabling neovascularization [153,154]. Fatahian et al. developed coaxially electrospun PLA nanofibers loaded with ibuprofen and ZnO nanoparticles, which exhibited over 90% antibacterial activity against *S. aureus* and *E. coli* and reduced TNF-α and IL-6 levels by more than 50% in LPS-stimulated macrophages, highlighting their dual antimicrobial and anti-inflammatory potential for chronic wound care [155]. Parangusan et al. demonstrated that electrospun PLA scaffolds incorporated with platelet-rich plasma (PRP) promoted M2 macrophage polarization by increasing IL-10 and TGF-β expression by around 2.5 fold while suppressing TNF-α and IL-6 by ~50%, leading to enhanced angiogenesis and accelerated wound closure in mouse models. These results support the use of PLA/PRP composites as immunomodulatory biomaterials for effective wound healing [156]. Overall, the chemical tunability of PLA and its capacity to serve as a delivery matrix for diverse therapeutic agents establish it as a highly adaptable and effective platform for advanced wound healing applications.

#### 2.2.2. Polyglycolic Acid: Crystallinity-Driven Biodegradability for Biomedical Scaffolds

Polyglycolic acid (PGA) is a linear aliphatic polyester with high crystallinity, belonging to the poly-α-hydroxy acid family [45,157,158]. It can be synthesized by polycondensation or ring-opening polymerization. Polycondensation typically occurs at 180–220 °C but is limited by water generation and solvent removal issues [159,160]. To overcome these limitations, Kricheldorf et al. reported that conducting polycondensation of glycolic acid in 1,2-dichlorobenzene at 180 °C for 120 h using p-toluenesulfonic acid (TSA) as a catalyst resulted in polyglycolic acid (PGA) with a melting enthalpy of 172.1 J g^−1^ and a crystallinity of 83%, indicating high molecular regularity and effective polymer chain growth under controlled dehydration conditions [160]. This study addresses the limitation of low molecular weight formation due to water accumulation during high-temperature polycondensation by optimizing thermal conditions and using a high-boiling solvent to facilitate dehydration. In another study, Sanko et al. reported that under azeotropic distillation conditions with trifluoromethanesulfonic acid (triflic acid) and anisole, polycondensation for 30 h yielded polyglycolic acid (PGA) with a number-average molecular weight (Mn) of 32,100 g mol^−1^ and a weight-average molecular weight (Mw) of 40,300 g mol^−1^, along with a hexafluoroisopropanol (HFIP) solubility of 147.3 mg mL^−1^, which is 3.7 times higher than that of commercial PGA, indicating significantly improved processability [161]. This strategy overcomes the challenge of tedious solvent and byproduct removal by employing azeotropic distillation, thereby enhancing both molecular weight and solubility for easy processing. In contrast, ring-opening polymerization of glycolide yields high MW PGA at milder temperatures (150–180 °C) with a tensile strength of 70–100 MPa and crystallinity > 45% [162,163,164]. It hydrolyzes into glycolic acid, a TCA-cycle-compatible metabolite, though local pH drops may occur during degradation [165,166]. Copolymerization plays a crucial role in overcoming degradation-related limitations of PGA. Little et al. reported that annealed PGA-co-butylene succinate (90% GA) reached Mw 97,000 g mol^−1^, with 7.2× tensile strength increase and 3.1× reduced elongation compared to its amorphous counterpart [158]. These tunable features support the utilization of PGA in short-term scaffolding or tissue regeneration in wound healing applications. For instance, Hadi et al. demonstrated that a wound scaffold composed of a polyglycolic acid (PGA) mesh and collagen sponge (PGAC) promotes wound healing and modulates inflammation in an animal model by leveraging the rapid biodegradability and high crystallinity-derived mechanical stability of PGA. The PGAC dressing functioned as an effective temporary biodegradable scaffold by enhancing compressive strength, inducing M2 macrophage polarization, and promoting angiogenesis [167]. Kakei et al. demonstrated in a rat dorsal skin wound model that application of a PGA sheet suppressed early-stage inflammation and modulated neo-epithelial tissue formation during wound healing. Histological analysis using Masson’s trichrome and α-SMA staining quantified tissue regeneration, while Iba-1 staining assessed inflammatory cell accumulation. The results showed that the PGA sheet, being biodegradable, temporarily reduced early inflammation; however, a slight increase in inflammatory cells was observed after day 12 [168]. This suggests that a balance between the transient mechanical support and in vivo reactivity of PGA must be carefully considered. This study suggests the potential of PGA as an effective wound support material by utilizing its high crystallinity and biodegradability.

#### 2.2.3. Poly (Lactic-co-Glycolic Acid): Tunable Copolymer for Controlled Drug Delivery

Poly(lactic-co-glycolic acid) (PLGA) is a copolymer of PLA and PGA. It is a representative biodegradable synthetic polymer belonging to the poly-α-hydroxy acid family. The chemical formula of PLGA is (C_3_H_4_O_2_)m-(C_2_H_2_O_2_)n, and its physical properties and degradation rate depend on the molar ratio of PLA:PGA [133,169,170]. The synthesis method is mainly synthesized via ring-opening polymerization (ROP). Lactide (PLA precursor) and glycolide (PGA precursor) are thermally ring-opened polymerized under a tin-based catalyst (e.g., stannous octoate) [171,172]. The reaction proceeds at 130–180 °C, and purification and decatalysis are essential to obtain high-purity PLGA. By adjusting the PLA:PGA ratio, stereochemistry, and molecular weight (Mw), various properties such as mechanical strength, flexibility, and degradation rate can be customized [173,174]. Increasing the PLA ratio increases the mechanical strength and slows down the degradation rate, while increasing the PGA ratio increases the water absorption capacity and speeds up the degradation rate [164,175]. PLGA is degraded by hydrolysis by water, and the degradation products are metabolized into the body’s lactic acid and glycolic acid. Accumulation of mono-acidic degradation products may cause a decrease in local pH and potential tissue irritation [176]. To overcome this limitation, Jang et al. reported that incorporating magnesium hydroxide (Mg(OH)_2_) particles, especially in fiber or whisker shapes, into PLGA significantly mitigated acidic degradation byproducts, maintaining a near neutral pH, reducing inflammatory cytokines (IL-6, IL-8), and improving cell proliferation and mechanical strength [177]. In another study, Jusu et al. loaded anticancer drugs into PLGA-PEG microspheres and applied them to triple-negative breast cancer, which resulted in increased cytotoxicity and inhibition of tumor recurrence with sustained release for about 62 days as well as improved survival in animal models [178]. This demonstrated that PEG blending effectively mitigated the problems of pH degradation and tissue irritation of PLGA. Due to its adjustable properties, PLGA is suitable for use as a drug delivery system and tissue engineering scaffolds in wound healing.

#### 2.2.4. Polycaprolactone: Durable Polymer for Tissue Support and Drug Delivery

Polycaprolactone (PCL) is a linear aliphatic polyester. The chemical formula of PCL is (C_6_H_10_O_2_)_n_, and its molecular structure contains relatively long methylene chains (-CH_2_-), which gives it high flexibility [179,180]. PCL is primarily synthesized via the ring-opening polymerization of ε-caprolactone in the presence of a catalyst. Commonly used catalysts include stannous octoate (Sn(Oct)_2_) and other organometallic compounds. Polymerization typically proceeds at temperatures ranging from 100 to 180 °C, with reaction times varying from a few hours to several tens of hours, depending on the desired molecular weight and reaction conditions [179,181,182]. Physical properties can be controlled by modifying the molecular weight (Mw) and end groups. PCL is a semi-crystalline polymer with high flexibility. It also has a low melting point (Tm ≈ 59–64°C), facilitating processing [180,183,184]. PCL is a slow-degradation polymer that slowly degrades by hydrolysis and enzymatic degradation. The degradation product, 6-hydroxyhexanoic acid, can be possible to metabolize in the body [185,186,187]. In a study, Asaduzzaman et al. demonstrated that PCL nanofibrous webs embedded with 1.9 wt% Candida antarctica lipase B (CALB) degraded completely within 15 min at 40 °C in a pH 8 buffer, whereas unmodified PCL did not degrade under the same conditions, highlighting the catalytic effect of entrapped enzymes in accelerating hydrolysis [188]. And, Suzuki et al. reported that PCL specimens immersed in seawater for 5 weeks lost all tensile strength along with ~34% of their weight, confirming PCL’s effective hydrolytic and enzymatic degradation in marine environments [186]. These properties make it a popular choice for treatments that require long-term support (e.g., artificial ligaments, nerve regeneration, etc.). In wound healing, it is considered a suitable material due to its long-lasting properties and the possibility of drug delivery due to its high hydrophobicity [189]. Furthermore, a summary of the types and advantages of both natural and synthetic biopolymers is presented in Table 2.

## 3. Biopolymeric Composites in Wound Healing

Recently, biopolymeric composites have gained extensive attention in wound healing applications due to their multifunctional capabilities. Beyond facilitating tissue regeneration, biopolymeric composites are suitable for monitoring wound sites and the general healing process. Moreover, the potential of biopolymeric composites as integrated platforms for both therapeutic intervention and diagnostic evaluation in wound management is increasing [195,196,197,198].

### 3.1. Types of Biopolymeric Composites and Their Applications in Wound Healing

Biopolymeric composites are multifunctional materials based on at least one biopolymer and composed of various reinforcing components. The combination of different biopolymers and reinforcing components can be used to tune physical, chemical, and biological functions [199,200]. Biopolymeric composites can overcome the limitations of using biopolymers alone. Especially, in the field of wound healing, biopolymeric composites can possess properties, such as elasticity, degradability, antimicrobial action, and drug delivery potential, making them a promising class of materials in the smart wound healing field [14,15,201]. Biopolymer composites can be customized into materials with different functions, depending on their combination. This review paper categorizes various biopolymer composites according to several classification criteria. These criteria are not absolute and can be merged or subdivided.

#### 3.1.1. Classification of Biopolymeric Composites by Matrix Type: Natural and Synthetic

The primary classification of biopolymeric composites is based on the nature of the polymer matrix, categorizing them into two distinct groups: those comprising natural polymer-based matrices and those incorporating synthetic polymer-based matrices.

**Figure 7:**
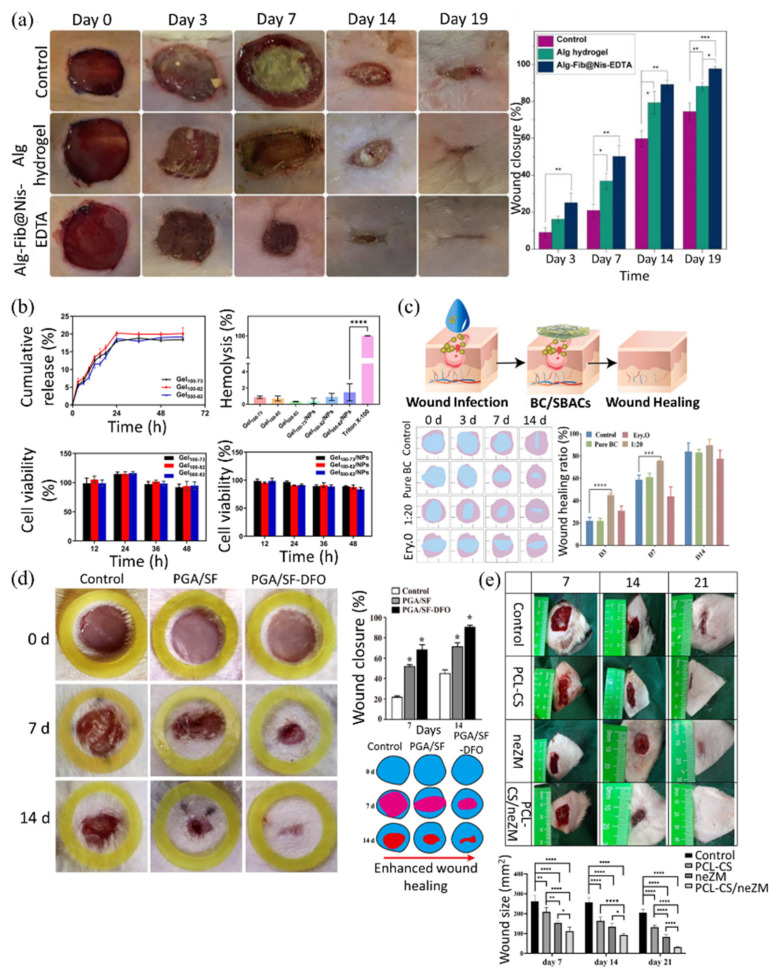
(**a**) Representative images of wound closure progression on days 0, 3, 7, 14, and 19 for control, alginate hydrogel, and Alg-Fib@Nis-EDTA-treated groups. The Alg-Fib@Nis-EDTA group showed markedly enhanced healing over time. Quantitative analysis of wound closure percentage, demonstrating significantly faster healing in the Alg-Fib@Nis-EDTA group, achieved 97.9% closure by day 14. Reprinted from [206] under Creative Commons License CC-BY 4.0. (**b**) Characterization of the nisin-loaded chitosan-based thermo-sensitive hydrogel system, demonstrating its physicochemical performance and biocompatibility. The hydrogel exhibited sustained nisin release over 24 h (~20%), minimal hemolysis (<2%) in sheep red blood cell assays, and maintained over 50% cell viability in RAW 264.7 macrophages for up to 48 h. These results confirm the system’s safety, hemocompatibility, and potential applicability as a short-term antibacterial wound dressing. Reprinted from [210] under Creative Commons License CC-BY 4.0. (**c**) In vivo evaluation of the wound healing efficacy of the BC/SBACs composite hydrogel in a rat full-thickness wound model. A schematic illustrates the infection treatment and healing process using the BC/SBACs dressing. Quantitative analysis showed significantly enhanced wound closure in the BC/SBAC (1:20) group by day 14, demonstrating the hydrogel’s therapeutic potential. Reprinted with permission from [217]. (**d**) In vivo evaluation of diabetic wound healing using electrospun PGA/SF scaffolds loaded with deferoxamine (DFO). Compared to the control and PGA/SF groups, the PGA/SF–DFO composite showed significantly accelerated wound closure (∼90.4% by day 14), as confirmed by photographic assessment, wound area quantification, and schematic visualization. Reprinted from [222] under Creative Commons License CC-BY 4.0. (**e**) Wound size quantification over 21 days shows significantly accelerated healing in the PCL–CS/neZM group compared to controls (*p*  <  0.05 to *p*  <  0.0001), supporting its regenerative potential. Reprinted from [226] under Creative Commons License CC-BY 4.0.

Firstly, the natural biopolymer-based matrices refer to composites based on natural biopolymers such as alginate, chitosan, hyaluronic acid, and cellulose. These naturally derived matrices are biocompatible and biodegradable, making them ideal materials for wound healing [202,203]. To elaborate further, alginate-based composites can be combined with polyvalent cations, such as Ca^2+^, to form hydrogels. The network structure inside the gel can be utilized for drug loading and release. They also have excellent water retention capabilities and have little inflammatory response. However, their ionic structure is unstable under physiological conditions, making them vulnerable to long-term application [204,205]. In a study by Soleimanpouran et al., it has been reported that alginate–fibrinogen hydrogel crosslinked with Ca^2+^ and loaded with 0.15 mM nisin and 50 mM EDTA exhibited high porosity (14–198 µm), a swelling ratio of ~2260%, and sustained nisin release (98% over 48 h). It showed strong antibacterial activity and enhanced wound closure (97.9% by day 14) with increased collagen deposition in vivo (Figure 7a) [206]. Chitosan-based composites are positively charged, which allows them to bind to negatively charged bacterial membranes and exhibit antimicrobial effects; they also induce platelet aggregation and allow drug attachment via amine groups (-NH_2_), and they are biodegradable. However, when used alone, they have low solubility at physiological pH (neutral) and weak mechanical strength [207,208,209]. In a recent study, Huang et al. developed a chitosan-based thermo-sensitive hydrogel embedding nisin-loaded nanoparticles (122.7 ± 4.9 nm; zeta potential: 23.9 ± 0.4 mV), achieving 86.2% encapsulation efficiency and 239.1 ± 7.2% swelling. The system released 20.1 ± 1.7% of nisin over 24 h and showed <2% hemolysis. Biocompatibility was confirmed via RAW 264.7 macrophage assays (viability > 50% up to 48 h) and hemolysis tests with sheep blood. It also exhibited strong antibacterial activity against *Staphylococcus aureus*, supporting its use as a short-term wound dressing (Figure 7b) [210]. HA-based composites have high water retention, provide a moist environment at the wound site, and are viscoelastic, forming into different sizes. Depending on their molecular weight, high molecular weight HA can be used to maintain tissue structure, while low molecular weight HA can be used for immunomodulation and cell stimulation. However, they can be easily degraded by enzymes (hyaluronidase), which can reduce their functional durability [211,212,213]. A hyaluronic acid-coated melt electro written scaffold functionalized with RGD peptides was shown to enhance C2C12 myoblast attachment, alignment along 10–20 μm fibers, and myotube formation; increased myosin heavy chain expression and peak creatine kinase activity on day 5 confirmed effective differentiation. Notably, hydrazone crosslinking of HA using norbornene and adipic acid dihydrazide significantly improved structural stability, maintaining hydrogel integrity for over 28 days in vitro and enhancing resistance to enzymatic degradation, as shown by Galindo et al. [214]. Finally, cellulose-based composites have good tensile strength and structural stability. They are biodegradable and have good water absorption and evaporation control, making them suitable for wound healing. They can also be processed with nanocellulose to increase surface area and improve drug adhesion. However, insolubility limits their use without surface modification [215,216]. To overcome this limitation, Xu et al. developed a bacterial cellulose (BC)-based hydrogel incorporating Scutellaria baicalensis extract (SBAC), rich in baicalin and baicalein. The resulting BC/SBAC composite showed enhanced mechanical strength, thermal stability, and biocompatibility. It achieved high drug loading (322 mg/g baicalin; 8.79 mg/g baicalein) and sustained release (~90% over 240 min). In vivo tests in rats confirmed accelerated wound healing, increased collagen deposition, and improved angiogenesis (Figure 7c) [217].

Second, synthetic biopolymer-based matrices refer to composites made from synthetic biopolymers such as polylactic acid (PLA), polyglycolic acid (PGA), poly (lactic-co-glycolic acid) (PLGA), and polycaprolactone (PCL). These synthetic polymer-based matrices mimic the properties of natural biopolymers. So, synthetic biopolymer-based matrices can also have a biocompatible and biodegradable function [135,218,219]. To elaborate further, PLA-based composites are biodegradable and biocompatible because they are based on lactic acid. They are also easy to process and possess high mechanical strength due to their versatility. However, they have low water absorption and lack flexibility [220]. To address these limitations, Heydari et al. incorporated platelet-rich plasma (PRP) into electrospun PGS/PLA nanofibers, which improved hydrophilicity (contact angle: 28.6°), swelling (187%), and flexibility due to the elastic nature of PGS, thereby overcoming the intrinsic water absorption and stiffness limitations of PLA. The composite enhanced fibroblast migration (99% wound closure at 24 h), collagen expression, and angiogenesis while modulating inflammation by reducing TNF-α and increasing IL-10, supporting its potential in wound healing [154]. PGA-based composites are biodegradable, have high mechanical strength, and are highly absorbent in acidic moisture [221]. A study by Zha et al. introduced electrospun nanofibrous scaffolds composed of PGA and silk fibroin (SF) loaded with deferoxamine (DFO) to enhance diabetic wound healing. The composite demonstrated controlled biodegradation (~20% mass loss over 14 days), high tensile strength (5.95 ± 0.13 MPa), and improved vascularization and collagen deposition. In vivo, it accelerated wound closure (approximately 90% by day 14) and eliminated the need for removal, reducing the risk of secondary tissue damage (Figure 7d) [222]. PLGA-based composites are copolymers of PLA and PGA with a controlled degradation rate by adjusting the ratio. They have excellent biocompatibility and can control the release of drugs [133]. In a recent study, Peng et al. developed stem-cell-membrane-coated PLGA-PEI nanoparticles loaded with miR-21, which enhanced wound healing by promoting fibroblast migration, collagen synthesis, and angiogenesis while reducing cytotoxicity commonly associated with PEI [223]. However, due to the presence of PGA, acidic degradation byproducts can adversely affect the wound site [166]. A study by Ruan et al. addressed this challenge by introducing alkaline piperazine segments into polyurethane–urea (P-PUU) scaffolds. This design effectively neutralized excess acidity, maintaining the interfacial pH near 8.1 and significantly enhancing osteoblast proliferation, differentiation, and extracellular mineralization [224]. PCL-based composites have a slow degradation rate, making them suitable for long-term support scaffolds. They are also highly flexible, elastic, and biocompatible. However, their slow degradation rate may not be suitable for acute wounds or treatments requiring rapid tissue regeneration. A PCL–chitosan nanofibrous scaffold coated with a gel containing *Zataria multiflora* nanoemulsions significantly accelerated wound closure and improved collagen deposition, re-epithelialization, and inflammation reduction over 21 days in a full-thickness rat wound model, as demonstrated by Osanloo et al. (Figure 7e) [225]. The experiment showed that the combination with chitosan improved water absorption and bioreactivity, compensating for the slow degradation of PCL.

In conclusion, biopolymeric composites have demonstrated strong potential in wound healing by overcoming the intrinsic limitations of individual biopolymers and synergistically enhancing biocompatibility, bioactivity, and therapeutic performance.

#### 3.1.2. Classification of Biopolymeric Composites According to the Reinforcement Type: Nanoparticle-Incorporated Composites, Fiber-Reinforced Composites, and Drug-Loaded Composites

The second classification is based on the type of reinforcement. According to this classification, there are three types of biopolymer composites: nanoparticle-incorporated composites, fiber-reinforced composites, and drug-loaded composites.

**Figure 8:**
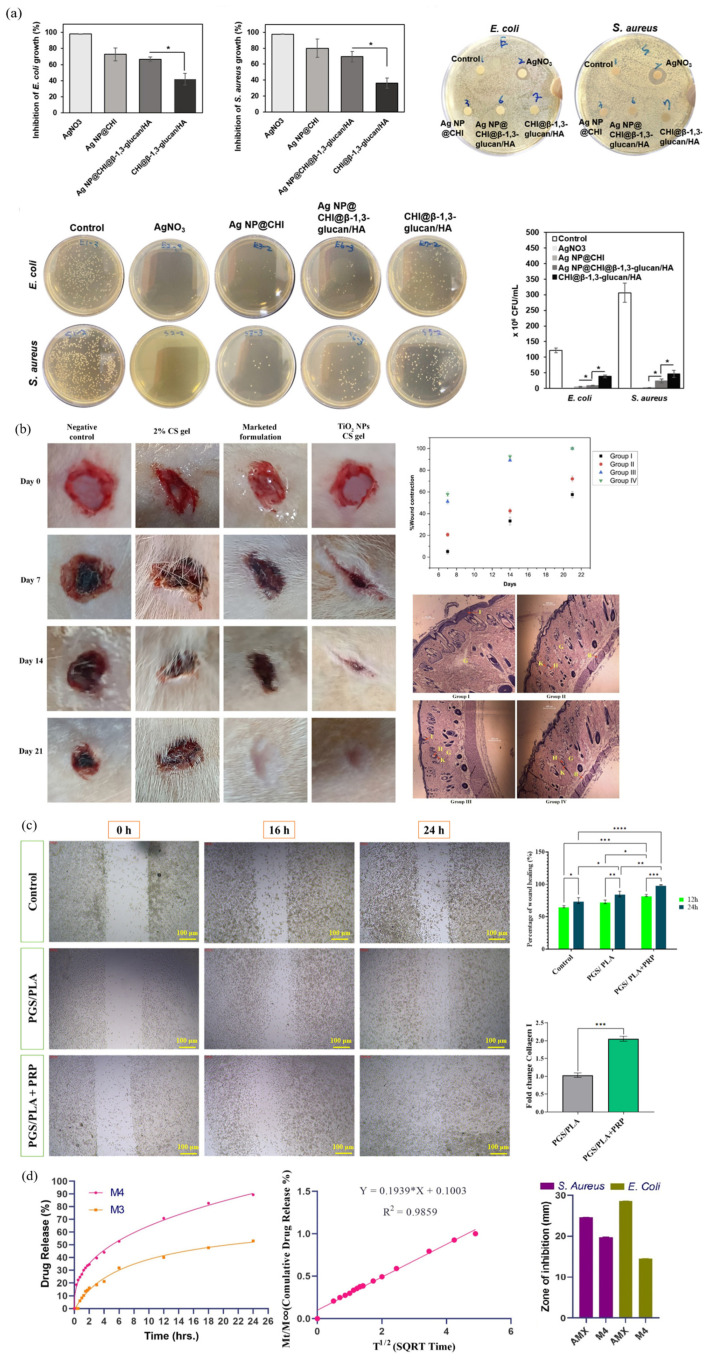
(**a**) Ag NP@CHI@β-1,3-glucan/HA exhibited strong antibacterial activity, inhibiting the growth of *E. coli* and *S. aureus* by 66–69% at 25 ppm Ag. This was demonstrated through limited colony formation and decreased CFU counts compared to controls, confirming the composite’s antimicrobial potential for wound healing applications. Reprinted from [230] under Creative Commons License CC-BY 4.0. (**b**) Macroscopic evaluation of diabetic wound healing over 21 days in rats shows that TiO_2_ NP–chitosan gel achieved complete wound closure by day 21, outperforming 2% CS gel and marketed silver sulfadiazine formulation. Reprinted from [231] under Creative Commons License CC-BY 4.0. (**c**) PGS/PLA nanofibers loaded with PRP promoted rapid fibroblast migration and collagen I expression. Scratch assay images show nearly complete wound closure at 24 h in the PGS/PLA+PRP group, with 99.05% healing. Collagen I expression was also significantly increased, supporting enhanced regenerative activity. Reprinted from [154] under Creative Commons License CC-BY 4.0. (**d**) The tannic acid-loaded M4 film exhibited sustained drug release, reaching 89.4% within 24 h. Antibacterial assays demonstrated strong inhibition against S. aureus (22.8 mm) and *E. coli* (18.6 mm), confirming the film’s effective release profile and antimicrobial potential. Reprinted from [6] under Creative Commons License CC-BY 4.0.

First, nanoparticle-incorporated composites refer to composites with inorganic nanoparticles such as silver nanoparticles (AgNPs), zinc oxide (ZnO), and titanium dioxide (TiO_2_) added to the matrix. Nanoparticles kill pathogens by destroying cell membranes or generating reactive oxygen species (ROS). They can also inhibit inflammatory responses and induce angiogenesis and fibroblast activation at wound sites [226,227,228,229]. Using AgNPs, Yao et al. developed a chitosan–silver nanocomposite integrated with β-1,3-glucan and hyaluronic acid, which demonstrated strong antimicrobial activity (inhibition of *E. coli* and *S. aureus* by 66–73% at 25 ppm Ag), excellent hemocompatibility (<5% hemolysis), enhanced coagulation (clotting time < 120 s), and significantly promoted fibroblast migration in scratch assays (68.6% closure at 8 h vs. 59% in control), confirming its wound healing potential (Figure 8a) [230]. In another study, titanium dioxide nanoparticles synthesized via a green method using Ocimum sanctum leaf extract were incorporated into a 2% chitosan gel and applied to diabetic wounds in rats. The TiO_2_–CS composite significantly accelerated wound closure, achieving complete healing within 21 days, and showed superior performance to 1% silver sulfadiazine cream. Histological analysis confirmed enhanced epithelialization, collagen deposition, and angiogenesis, with reduced inflammation, demonstrating its therapeutic potential for chronic wound healing, by Ahmad et al. (Figure 8b) [231]. Both studies demonstrate that incorporating metal-based nanoparticles into biopolymer matrices can significantly enhance antimicrobial efficacy, biocompatibility, and wound healing outcomes. However, high concentrations of nanoparticles can be lethally toxic to cells. Therefore, quantitative control and controlled release are required.

Second, fiber-reinforced composites refer to composites with fibers such as electrospun nanofibers, collagen fibers, and synthetic polymer fibers added to the matrix. Fibers add structural support. They increase tensile strength and increase surface area, allowing the composite to remain stable over the wound. Fiber diameter, arrangement, etc., can be adjusted to control exudate drainage and oxygen exchange [153,232,233]. In a recent study, Dong et al. developed fiber-reinforced biopolymeric composites using recombinant human-like collagen (RHC) and fibronectin (rhFN), which enhanced dermal fibroblast adhesion, migration, and proliferation in vitro. In a full-thickness skin defect model, wounds treated with the RHC/rhFN scaffold achieved significantly faster closure (73.3 ± 7.1% by day 3) compared to controls (52.6 ± 3.1%), with improved epithelial thickness, organized collagen deposition, and increased angiogenesis, confirming the structural and regenerative benefits of fibrous ECM-mimetic reinforcement for wound healing [234]. In another study, electrospun PGS/PLA nanofibers loaded with platelet-rich plasma (PRP) significantly enhanced wound healing by improving hydrophilicity (contact angle: 28.6°), promoting fibroblast migration (99.05% closure at 24 h), and increasing collagen I expression and angiogenesis (Figure 8c) [153]. This experiment demonstrates the structural and biological advantages of nanofiber reinforcement. However, the complexity and high cost of the fabrication process, along with potential delamination at the fiber–matrix interface, pose significant challenges that must be addressed to ensure practical applicability.

Lastly, drug-loaded composites refer to composites with therapeutic agents, such as antibiotics, anti-inflammatories, growth factors, etc., inserted or bound directly within the polymer. They can induce sustained or stimuli-responsive therapeutic release to the wound site to inhibit infection and induce regeneration [235,236,237,238]. They can be designed as a sustained release type, which allows the drug to be released steadily over a period, or as a stimuli-responsive release type, which allows the drug to be released selectively in response to pH changes, temperature changes, enzyme presence, etc. Depending on the type and amount of therapeutic agent loaded, antimicrobial effects or regeneration-inducing effects can be achieved [239,240,241]. In a recent study, Ali et al. developed a chitosan–carboxymethyl cellulose film loaded with tannic acid, which exhibited 89.4% drug release over 24 h and strong antibacterial activity (80.8% against *S. aureus*). In a rabbit wound model, the film accelerated healing to over 88% by day 7 and achieved complete skin regeneration within 10–12 days, demonstrating its efficacy in promoting rapid wound repair and infection control (Figure 8d) [6]. In another study, Mahmood Faal et al. fabricated electrospun PLA nanofiber dressings loaded with curcumin and carbon nanotubes (CNTs) to achieve sustained drug delivery and improved wound healing efficacy. The composite with 0.03% CNTs demonstrated optimal performance, with controlled curcumin release sustained over 168 h, enhanced antibacterial activity (78.17% inhibition against *S. aureus*), and improved tensile strength (2.66 MPa). Cytotoxicity tests using L929 cells confirmed high biocompatibility over 7 days, and SEM imaging showed strong cellular adhesion. These results confirm the potential of composites as a therapeutic wound dressing with effective drug release, mechanical support, and antimicrobial function [242]. Drug-loaded biopolymeric composites offer promising therapeutic benefits, but the stability of encapsulated agents and real-time responsiveness to changing wound conditions require further improvement for optimal performance.

Collectively, reinforcement in biopolymer composites offers significant benefits to wound healing by enhancing antimicrobial activity, structural support, and controlled drug delivery.

## 4. Future Perspectives and Conclusions

Biopolymeric composites are the best solution in the field of wound healing, but several challenges still exist that are restricting their widespread clinical application and commercialization. The following future perspectives presented here will help to address these challenges and make biopolymer composites one of the most promising materials for wound healing.

**Enhanced long-term stability under physiological conditions.** Biopolymer complexes lack long-term stability in physiological environments. For example, ionic hydrogels that use divalent cations, such as alginate/Ca^2+^, can undergo ion exchange with other monovalent cations in the body, resulting in structural collapse. They can also be irreversibly degraded by enzymes, such as hyaluronidase, cellulase, and chitinase, limiting their use as drug delivery or tissue scaffolds [243,244]. In experiments by Velasco-Rodriguez et al., hybrid hydrogels using methacrylated gelatin (GelMA) and methacrylated hyaluronic acid (HAMA) were reported to have improved resistance to degradation by collagenase. These photoinitiated crosslinked matrices can be tailored to customize mechanical properties and degradation rates [245]. In addition, additional resistance to ion exchange and enzymatic degradation can be achieved by introducing nanoreinforcements such as natural–synthetic polymeric interwoven networks (semi-IPNs), graphene oxide, and cellulose nanocrystals [246,247]. These design strategies should be explored to increase long-term stability by delaying the collapse of the composite structure, even after prolonged exposure to changes in ionic conditions commonly present in chronic wound environments.

**Balancing Mechanical Strength and Biodegradability.** For the long-term and efficient use of composites, a balance between mechanical strength and biodegradability is essential. Natural polymers with high biodegradability have low mechanical strength. Synthetic polymers have high mechanical strength but low biodegradability, meaning they degrade slowly over a long period of time, which can lead to acidic byproducts or tissue irritation [45]. In response to this, future composite design strategies should be directed toward satisfying both the mechanical durability and biodegradability of composites. For example, bilayer electrospun fiber nets composed of a polyurethane (PU) top layer and a PCL/gelatin bottom layer have been shown to have dramatically improved structural strength and stability compared to single materials [248]. In addition, ester-based hydrolyzable crosslinkers should be introduced to the synthetic polymer chain to control the rate of degradation, or buffers, such as Mg(OH)_2_, should be added to mitigate the effects of acidic byproducts of degradation [249,250]. These multilayer structures or crosslinker-controlled designs help to ensure that the composites are biodegradable while maintaining long-term physical robustness.

**Standardization and Scale-Up of Biopolymer Production.** Biopolymer composites are difficult to produce in large quantities and, especially for natural biopolymers, properties such as the degree of deacetylation and molecular weight can vary, depending on the extraction route and purification feedstock and method. This may cause inconsistencies in the biological properties of the composites. Properties such as degradation rate, viscosity, or bioactivity may be inconsistent [251,252]. Fermentation methods using microorganisms from the genus *Streptococcus zooepidemicus* or *Komagataeibacter* are gaining traction. For example, *Streptococcus zooepidemicus* is easy to culture and can produce polymeric HA in large quantities while synthesizing pure HA without toxic chemicals. Meanwhile, bacteria in the genus *Komagataeibacter* produce large quantities of biocellulose, which is higher in purity than vegetable cellulose [253,254]. By utilizing these biotech fermentation systems, biopolymers of uniform quality can be reliably obtained under the same conditions. At the same time, regulatory approval and industrialization require standardization of quality control metrics such as molecular weight distribution, dissolution, and bioactivity.

**Advancing Clinical Validation and Commercial Viability.** Clinical validation and commercialization are challenging processes. Despite the success of many in vitro and in vivo experiments, biopolymeric composites remain stuck in the lab. There are limitations to assessing long-term stability in real patient populations, and the complexity and high cost of manufacturing are also barriers to commercialization. To overcome these barriers, preclinical experiments need to be intensified in a variety of wound morphologies in different animal models. In addition, modular and scalable manufacturing platforms, such as automated film casting and continuous hydrogel printing, should pave the way for mass production. Finally, public–private partnerships and regulatory science research must be strengthened to accelerate product approval and market entry [255,256].

In conclusion, it is understood that by combining biological compatibility, controllable degradation, and multifunctional therapeutic properties, biopolymeric composites have become highly promising for next-generation wound healing materials, which can efficiently overcome the limitations of traditional dressings. By blending natural polymers such as chitosan, alginate, hyaluronic acid, and cellulose with synthetic polymers, like PLA, PLGA, PGA, and PCL, these composites can be precisely engineered to address the specific and dynamic requirements of various wound types. Their response to physiological stimuli, including changes in pH, temperature, and bacterial infection, places them at the forefront of smart biomaterials that enable adaptive healing, antimicrobial protection, and targeted drug delivery. The integration of reinforcing components such as nanoparticles, electrospun fibers, and bioactive agents further expands their functional capabilities, facilitating simultaneous management of infection control, inflammation modulation, tissue regeneration, and scar reduction. Categorizing these materials based on their matrix composition and reinforcement strategies establishes a clear framework for deliberate material design aimed at specific therapeutic goals. Preclinical studies consistently demonstrate that biopolymeric composites outperform single-component systems, exhibiting improved wound closure rates, enhanced angiogenesis, increased collagen deposition, and accelerated re-epithelialization.

Despite these advances, critical challenges persist in translating biopolymeric composites into clinical applications. Foremost is the issue of long-term stability under physiological conditions, as many naturally derived hydrogels are vulnerable to degradation through ion exchange or enzymatic activity, leading to early loss of structural integrity. Future developments must focus on creating crosslinked hybrid networks, semi-interpenetrating polymer systems, or incorporating nanofillers that reinforce mechanical strength while maintaining biodegradability.

Balancing mechanical durability with biodegradability remains a significant concern. Although synthetic polymers contribute strength and controlled degradation, they may produce acidic degradation byproducts or lack inherent bioactivity. The next generation of composites should integrate functional additives such as buffering agents, enzymatically cleavable linkers, and bioactive peptides to mitigate these issues while enhancing therapeutic synergy. Scalability and reproducibility in manufacturing also present major hurdles. The variation in natural polymer sources, batch-to-batch inconsistencies, and complex fabrication techniques impede clinical standardization. Employing biotechnological methods, such as microbial fermentation for controlled production of hyaluronic acid or bacterial cellulose, offers a sustainable and scalable approach. Moreover, developing modular, automated fabrication platforms will be essential to reducing production costs and ensuring batch uniformity. Finally, while extensive in vitro and in vivo evidence supports the effectiveness of biopolymeric composites, robust clinical validation is still limited. Broader preclinical evaluations across diverse wound models and well-designed clinical trials are imperative to establish safety, efficacy, and economic viability. Accelerated translation from research to clinical practice will require close interdisciplinary collaboration among material scientists, clinicians, and regulatory agencies. In summary, biopolymeric composites mark a groundbreaking leap in wound care, offering tailored, adaptive, and multifunctional solutions. Continued advancements in material design, manufacturing techniques, and clinical application will be required to fully realize their promise in regenerative medicine and intelligent therapeutic systems.

## Figures and Tables

**Table 1:** A summary of commonly used synthetic biopolymers and their functional roles in various phases of the wound healing process.

Name	Chemical Structure	Properties	Ref.
Polylactic acid (PLA)	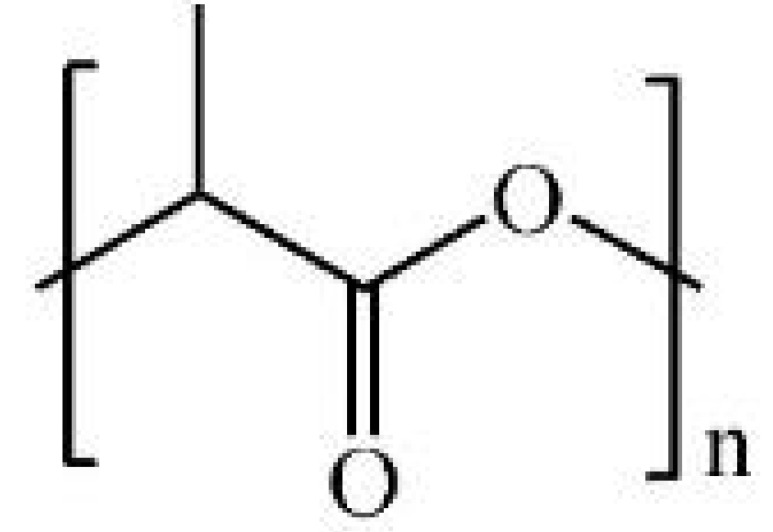	Electrospun mat mimics ECM Supports fibroblast/keratinocyte growth Dual antimicrobial and anti-inflammatory effects (contains ZnO/ibuprofen)	[122,123,124,125,126,127,128,129,130,131,132,133,134,135,136,137,138,139,140]
Polyglycolic acid (PGA)	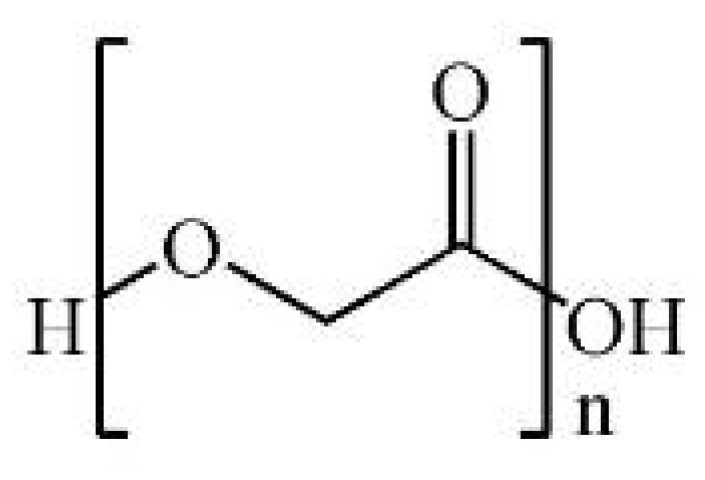	Acts as a temporary scaffold due to rapid biodegradability Combination with collagen (PGAC) reduces inflammation and induces angiogenesis Regulates neoepithelial formation and suppresses initial inflammation	[141,142,143,144,145,146,147,148,149,150,151,152]
Poly(lactic-co-glycolic acid) (PLGA)	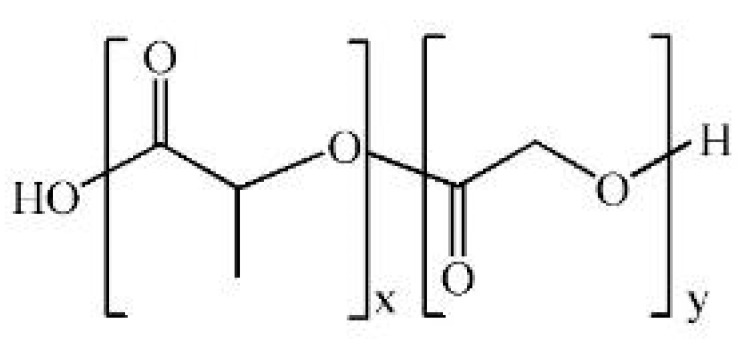	Adjustable degradation rate (PLA:PGA ratio) Mitigation of acidic byproducts with Mg(OH)_2_ or PEG Suitable as a drug delivery scaffold	[153,154,155,156,157,158,159,160,161,162]
Polycaprolactone (PCL)	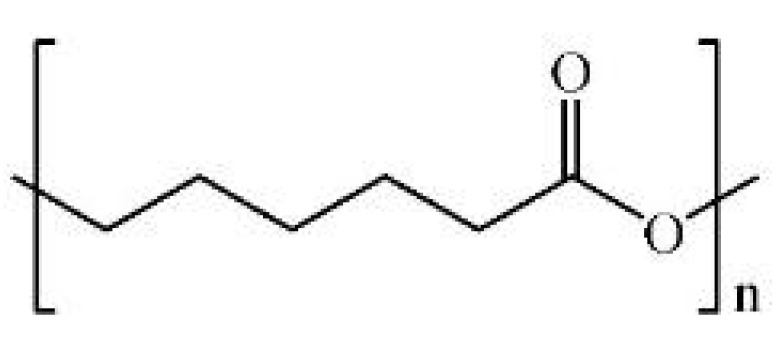	Provides high flexibility and long-term support structure Rapid degradation is possible when enzymes are loaded Supports long-term wound healing High water repellency → advantageous for drug delivery	[163,164,165,166,167,168,169,170,171,172,173]

**Table 2:** Classification of natural and synthetic biopolymers with representative examples and associated advantages.

Type	Examples	Advantages	Ref.
Synthetic biopolymers	Biosynthetic (microbial production): polyhydroxyalkanoates (PHA), polylactic acid (PLA) Chemically modified natural polymers: cellulose acetate, starch blends Fully synthetic biodegradable polymers: polyglycolic acid (PGA), polycaprolactone (PCL)	Tailorable properties (mechanical strength, degradation rate, thermal stability) Consistent quality and purity Scalable industrial production Can be engineered for specific applications (e.g., medical implants, packaging) Often more durable in specific environments	[45,190,191]
Natural biopolymers	Polysaccharides: cellulose, starch, chitin Proteins: collagen, keratin, silk Nucleic acids: DNA, RNA	Renewable and biodegradable Biocompatible (safe for medical use) Often non-toxic Derived from abundant natural sources Can have inherent biological activity	[192,193,194]

## Data Availability

The data will be available from the corresponding authors upon reasonable request.
